# Vibrational analysis of double-walled silicon carbide nano-cones: a finite element investigation

**DOI:** 10.1038/s41598-024-55536-1

**Published:** 2024-03-01

**Authors:** S. Nickabadi, R. Ansari, B. Golmohammadi, P. Aghdasi

**Affiliations:** 1 Faculty of Mechanical Engineering, University of Imam Khomeini Marine Sciences, Nowshahr, Iran; 2 Faculty of Mechanical Engineering, University of Guilan, P.O. Box 3756, Rasht, Iran; 3 Department of Physical Chemistry, Faculty Chemistry, University of Tabriz, Tabriz, Iran

**Keywords:** DW silicon carbide nano-cones, Vibrational properties, FEM, Length, Apex angle, Theory and computation, Mechanical engineering

## Abstract

A three-dimensional finite element model is used to investigate the vibrational properties of double-walled silicon carbide nano-cones with various dimensions. The dependence of the vibrational properties of double-walled silicon carbide nano-cones on their length, apex angles and boundary conditions are evaluated. Current model consists a combination of beam and spring elements that simulates the interatomic interactions of bonding and nonbonding. The Lennard–Jones potential is employed to model the interactions between two non-bonding atoms. The fundamental frequency and mode shape of the double-walled silicon carbide nano-cones are calculated.

## Introduction

Considering the excellent properties of silicon carbide (SiC), such as high mechanical strength, thermal conductivity, hardness as well as good radiation resistance, biological compatibility and electrical properties^1,2^ have made a potential candidate to be used in different fields such as microelectronics, nanoelectronics, photonics, biological imaging, energy storage, conversion, and medical science^3,4^. Theoretical methods used for investigating the physical properties of the nano-structures, can be generally divided into two different categories, atomistic approaches and continuum mechanics approaches. Common atomistic approaches include the classical molecular dynamics (MD) simulations^5^, tight-binding MD simulations^6^ as well as density functional theory (DFT)^7–15^. The continuum models also can be divided as Bernoulli–Euler and Timoshenko beam models^16–18^, shell models^19–21^ and space frame models^22–25^. Moon et al.^26^ showed that two types of silicon carbide nanotubes (SiCNTs) exist, type 1, where each Si atom is connected to three C atoms and vice versa and type 2, where each C atom is bonded to two Si atoms. The mechanical properties of SiCNTs were obtained with MD simulations. The elastic modulus of the SiCNTs has been determined as 650 GPa. Using the MD simulation method, Setoodeh et al.^27^ studied the buckling behavior of perfect and defected SiCNTs. The effects of tube diameter and chirality on Young’s modulus of SiCNTs showed Young’s modulus of SiCNT is almost independent from its diameter. Wang et al.^28^ and Makeev et al.^29^ investigated the mechanical properties of the SiC nanowires with MD simulations and the elastic properties of the 3C-SiC nanowires with different diameters under external loading have been calculated. It was found that SiC nanowires have high strength and high elastic modulus. Pan and Si^30^ studied the response of single crystalline SiCNTs under tensile strain with MD simulations. It has been found that the deformation proceeds only from bond-stretching and breaking. Furthermore, the brittle pattern was observed for the SiCNTs. Also, they have demonstrated that thickness and inner diameter respectively increase and decrease the mechanical behavior of SiCNTs. An accurate spring-mass model based three-dimensional finite element (FE) approach was used by Ansari et al.^31,32^ to predict the mechanical properties and analyze the vibration behavior of armchair and zigzag single-walled carbon nanotubes (SWCNTs). They used a rotational spring for describing the bond angle bending and out of plane angle torsion interaction. It was found that with the increase of the aspect ratio, the fundamental natural frequency of the SWCNTs would decrease. Furthermore, a significant dependence of the mechanical properties of armchair and zigzag SWCNTs on the nanotube radius was observe. Khani et al.^33^ studied the vibrational behavior of SiCNTs using a molecular mechanics-based FE method. The mode shapes and natural frequencies of the SiCNTs with different lengths, diameters, chiralities and boundary conditions were determined. The results indicated that the SiCNTs with clamped-free boundary conditions has smaller natural frequencies than those with the clamped–clamped boundary conditions. Ru^33^ used a continuum model allowing for the van der Waals (vdW) interlayer interactions to investigate the vibration of tight nanosprings. They investigated the influences of pitch, stiffness and the number of the nanosprings on the period and amplitude of the vibration. Although many researches have been done on investigating the physical properties of the single-walled nano-cones, there are limited studies about the multi-walled nano-cones. The vibrational behavior of close-tip multi-walled CNCs was investigated by Narjabadifam et al.^34^ using MD simulation. They extracted the resonant frequencies and their corresponding three-dimensional mode shapes using the atomic motions during the equilibrating process of the nanostructure and also found that the resonant frequencies depend on the apex angle and shape of the modal displacement. Employing the first-principles calculations, Brito et al.^34^ investigated the stability and electronic structure of DWCNCs 60°60°, 120°120° and 60°120° under compressive strain with several rotation angles between the walls. In another study Brito et al.^35^ used first-principle calculations to study the structural and electronic properties of different configurations of double-walled boron nitride nano-cones with a disclination angle of 60°. It was found that the non-rotated configuration of DWCNCs with a defective line composed by C and N atoms, forming C–N bonds are most stable. Ghorbanpour and Kolahchi^35^ used Eringen’s nonlocal theory and Timoshenko beam model to study the nonlinear vibration and instability of embedded DWCNCs subjected to axial load on the Winkler -Pasternak elastic medium. Recently, the FE method has been utilized to explore the vibrational characteristics of single-walled carbon nano-cones (SWCNCs) and double-walled carbon nano-cones (DWCNCs) by Gajbhiye and Singh^36^. It was shown that the fundamental natural frequency of the nano-cones is inversely affected by increasing their length.

After a comprehensive search, no thorough study was found on the physical properties of nano-cones including Si and C atoms. Therefore, the current article is aimed to study the vibrational properties of DW SiC nano-cones. In this article, the vibrational behavior on the double-walled silicon carbide nano-cones (DWSiCNCs) is investigated using nano-scale continuum mechanic’s approach. The fundamental natural frequencies and mode shapes of DWSiCNCs under various boundary conditions, apex angle and length are calculated. By applying the beam and spring elements, a FE modeling approach is utilized to achieve mode shapes and fundamental natural frequencies of DWSiCNCs. Current modeling approach can be extended to study other physical properties of the multi-walled SiC nano-cones such as their elastic properties. The findings from this study contribute to the understanding of the mechanical properties and behavior of DWSiCNCs as well as the effectiveness of the employed FE method in evaluating the natural frequencies and mode shapes of these nanostructures.

## Finite element formulation

### Covalent interactions

Molecular mechanics-based FE analysis is used to investigate the vibrational behavior of DWSiCNCs. DWSiCNCs are treated as a spaceframe structure in which consists a combination of beam and spring elements that simulates the interatomic interactions of bonding and nonbonding, respectively. To calculate the diameter and elastic and shear modulus of the beam elements, a linkage between the molecular mechanics and continuum mechanics is used. The term that expresses the total potential energy of a molecular system as the sum of bonded and nonbonded interatomic interactions energies is as follows^37^:

1 $$E = \sum U_{r} + \sum U_{\theta } + \underbrace {{\sum U_{\emptyset } + \sum U_{\omega } }}_{{U_{\tau } }} + \sum U_{vdW}$$

where $U_{r}$, $U_{\theta }$, $U_{\emptyset }$, $U_{\omega }$ and $U_{vdW}$ are energies corresponding to the bond stretching, bond angle bending, dihedral angle torsion, out-of-plane torsion and nonbonded van der Waals interaction, respectively.

Assuming small deformation and combining dihedral angle torsion ($U_{\emptyset }$) and out-of-plane torsion ($U_{\omega }$) into a single equivalent term, then the following terms are used to describe the potential energies^38,39^:

2 $$U_{r} = \frac{1}{2}k_{r} \left( {\Delta r} \right)^{2} , \frac{{d^{2} U_{r} }}{{d\Delta r^{2} }} = k_{r}$$

3 $$U_{r} = \frac{1}{2}k_{\theta } \left( {\Delta \theta } \right)^{2} , \frac{{d^{2} U_{\theta } }}{{d\Delta \theta^{2} }} = k_{\theta }$$

4 $$U_{\tau } = U_{\emptyset } + U_{\omega } = \frac{1}{2}k_{\tau } \left( {\Delta \emptyset } \right)^{2} , \frac{{d^{2} U_{\tau } }}{{d\Delta \tau^{2} }} = k_{\tau }$$

where $k_{r}$, $k_{\theta }$ and $k_{\tau }$ denote the bond stretching, bond angle bending and bond torsion force constants, respectively. While $\Delta r$, $\Delta \theta$ and $\Delta \emptyset$ indicate the bond stretching increment, the bond angle change and the angle of bond twisting, respectively.

The strain energy of a uniform beam under pure tension $N$, pure bending moment $M$ and torsion of beam element under torque $T$, are expressed as:

5 $$U_{A} = \frac{1}{2}\mathop \int \limits_{0}^{L} \frac{{N^{2} }}{EA}dL = \frac{1}{2}\frac{{N^{2} L}}{EA} = \frac{EA}{{2L}}\left( {\Delta L} \right)^{2}$$

6 $$U_{M} = \frac{1}{2}\mathop \int \limits_{0}^{L} \frac{{M^{2} }}{EI}dL = \frac{2EI}{L}\alpha^{2} = \frac{EI}{{2L}}\left( {2\alpha } \right)^{2}$$

7 $$U_{T} = \frac{1}{2}\mathop \int \limits_{0}^{L} \frac{{T^{2} }}{GJ}dL = \frac{1}{2}\frac{{T^{2} L}}{GJ} = \frac{GJ}{{2L}}\left( {\Delta \beta } \right)^{2}$$

where $L$ and $A$ are the beam length and cross-sectional area, $I$ and $J$ are the moment of inertia and polar moment of inertia of beam elements, $E$ and $G$ are the Young’s modulus and shear modulus and $\Delta L$, $2\alpha$ and $\Delta \beta$ are the axial stretching deformation, the total relative rotation angle and the relative torsion of beam ends, respectively. From equalization of the strain and potential energies in structural and molecular mechanics, the following relationships between the structural mechanics parameter $EA$, $EI$ and $GJ$ in Eqs. (5)–(7) and the molecular mechanics parameters $k_{r}$, $k_{\theta }$ and $k_{\tau }$ in Eqs. (2)–(4) can be achieved:

8 $$\frac{EA}{L} = k_{r}$$

9 $$\frac{EI}{L} = k_{\theta }$$

10 $$\frac{GJ}{L} = k_{\tau }$$

By simplifying the above equation, and assuming circular cross-section for the beam elements, the properties of the three-dimensional beam elements are calculated as follows^40–42^:

11 $$d = 4\sqrt {\frac{{k_{\theta } }}{{k_{r} }}} , E = \frac{{k_{r}^{2} L}}{{4\pi k_{\theta } }}, G = \frac{{k_{r}^{2} k_{\emptyset } L}}{{8\pi k_{\theta } }}$$

The values of force constants of Eqs. (2)–(4) are equal to $k_{r} = 417.156 \times 10^{ - 7} \;{\text{nN}}/{\text{nm}}$,$k_{\theta } = 0.842\;{\text{nN}}.{\text{nm}}$ and $k_{\tau } = 1.505\,{\text{nN}}.{\text{nm}}$, respectively^43^. After introducing these values into Eq. $\left( {11} \right)$ along with covalent bond distance of the carbon atoms $L = 1.80$ Å^44^ one could have *d* = 1.7971 Å, *E* = 2.9372 × 10^−8^ N/Å, *G* = 2.6256 × 10^−9^ N/Å

In the vibrational behavior of Double-Walled Silicon Carbide Nanotubes (DWSiCNCs) using molecular mechanics-based finite element analysis the nanotubes are modeled as a spaceframe structure composed of beam and spring elements, representing bonding and nonbonding interactions^45,46^. The total potential energy of the system is calculated by considering bond stretching, bond angle bending, bond torsion, and nonbonded van der Waals interactions^47,48^. The relationships between structural and molecular mechanics parameters are established, and simplified equations are provided to calculate properties such as diameter, Young's modulus, and shear modulus of the beam elements^49,50^. The analysis provides insights into the vibrational properties of DWSiCNCs, contributing to the understanding of their behavior and potential applications.

### Noncovalent interactions

Lennard–Jones (L–J) potential describes noncovalent interactions between atoms in a nano-cone structure^51,52^. The L-J ‘6–12’ potential function, defined by Eqs. (12) and (15), quantifies these interactions based on parameters such as well depth (ε), collision diameter (σ), and distance between atoms (*r*_*vdW*_). The equations also mentions the use of linear spring elements to model van der Waals interactions between different walls of the nano-cone. The stiffness of these spring elements is determined by the second derivative of the L–J potential function. The passage emphasizes the computational efficiency achieved by using the spring elements only for atoms within a certain distance threshold. The L–J ‘6–12’ Potential function is expressed as:

12 $$U_{vdW} = 4\varepsilon \left[ {\left( {\frac{\sigma }{{r_{vdW} }}} \right)^{12} - \left( {\frac{\sigma }{{r_{vdW} }}} \right)^{6} } \right]$$

$\varepsilon$ and $\sigma$ are the Lennard–Jones parameters, where $\varepsilon$ represents the well depth of the potential (energy parameter), $\sigma$ is defined as the collision diameter between two atoms. Furthermore, $r_{vdW}$ is the distance between the interacting atoms.

The Lennard–Jones parameters between the atoms $i$ and $j$ are obtained by the following relations:

13 $$\sigma_{ij} = \frac{{\sigma_{i + } \sigma_{j} }}{2}$$

14 $${\epsilon }_{ij=\sqrt{{\epsilon }_{i}{\epsilon }_{j}}}$$

Table 1 shows the L–J ‘6–12’ Potential function parameters for Si–Si, Si–C and C–C bonds^53^.

**Table 1:** Values of the L-J potential function parameters^53^.

Parameter	Si–Si	Si–C	C–C
$\sigma$(${\text{\AA}}$)	2.2951	1.7400	1.4806
$\varepsilon$(${\text{eV}}$)	2.817	3.895	5.437

**Figure 1:**
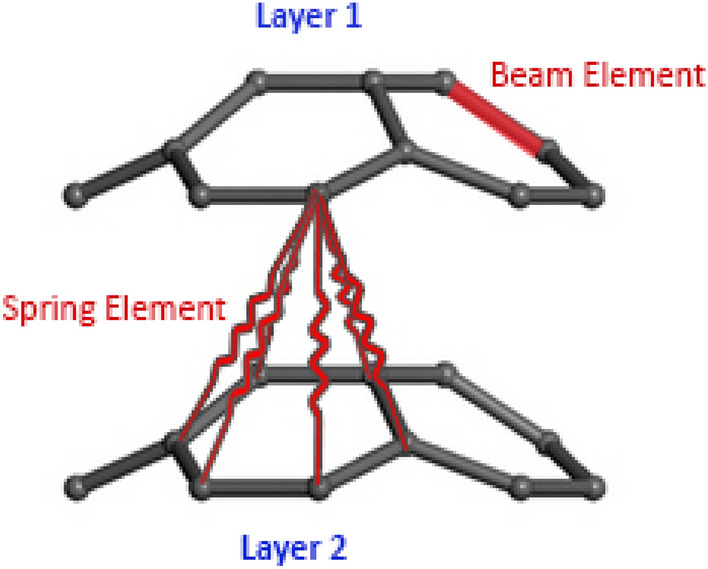
Modeling of beam element and spring element.

Besides, the linear spring elements are used for modeling the van der Walls interactions between different walls (Fig. 1). Calculating the second derivative of L–J ‘6–12’ potential function with respect to the distance between two atoms, the stiffness of these elements are obtained:

15 $$\frac{{d^{2} U_{vdW} }}{{dr_{vdW}^{2} }} = k_{vdW} = 4\varepsilon \left( {156\frac{{\sigma^{12} }}{{r_{vdW}^{14} }} - 42\frac{{\sigma^{6} }}{{r_{vdW}^{8} }}} \right)$$

To reduce the required computational resource, the spring elements are used between the atoms with the distances lower than $2.5\sigma$^54^. In summary, limitations and assumptions of the current modelling approach are as follows:

1. The nanostructure is considered as a defect-free structure.
2. The small deformations are considered for the structure and large deformations are ignored.
3. The harmonic form is considered for the strain energies in the molecular mechanics.
4. The atomic bonds are simulated by the beam elements.

Furthermore, as the model is developed based on the small deformation assumption, it cannot be used for the large deformations.

### Finite element formulation of the nanostructures vibrational analysis

The application of structural dynamics theories to study the behavior of structures is formulate for FE in this section. The equations explain the motion, which describes how a structure responds to external forces, and emphasizes the simplification achieved by neglecting the damping term. The modification of the mass matrix to a diagonal structure through lumped masses is described, as well as the consideration of stiffness matrices for beam and spring elements. The solution of the eigenvalue problem using the Block Lanczos method to determine natural frequencies and mode shapes is discussed. Finally, it highlights the conversion of natural frequencies to hertz, providing a concise overview of the key concepts and processes involved in analyzing structural dynamics.

Using the structural dynamics theories, the equation of motion of a structure can be written as^55^:

16 $$\left[ M \right]{ }\left\{ {\ddot{q}} \right\} + \left[ C \right]{ }\left\{ {\dot{q}} \right\} + \left[ K \right]{ }\left\{ q \right\} = \left[ F \right]$$

where ${ }\left[ M \right]$, $\left[ K \right]$,${ }\left[ C \right]$, $\left\{ q \right\}$,${ }\left\{ {\dot{q}} \right\}$ and $\left\{ {\ddot{q}} \right\}$ are the global mass matrix, global stiffness matrix, global damping matrix, nodal displacement vector, nodal velocity vector and nodal acceleration vector, respectively. It has been proved that the second term of Eq. (16) which is related to the damping can be neglected as compared to the other terms^56–58^. Therefore, Eq. (16) is converted to the following relation:

17 $$\left[ M \right]{ }\left\{ {\ddot{q}} \right\} + \left[ K \right]{ }\left\{ q \right\} = 0{ }$$

where ${ }\left[ M \right]$, $\left[ K \right]$, $\left\{ q \right\}$ and $\left\{ {\ddot{q}} \right\}$ are the global mass matrix, global stiffness matrix, nodal displacement vector and nodal acceleration vector, respectively. To have a diagonal mass matrix, a discrete point mass is located at each node. The resulting mass matrix is a diagonal mass matrix that is named as “lumped mass matrix”^59,60^. The global mass matrix is considered as follows:

18 $$\left[ M \right] = \left[ {\begin{array}{*{20}c} {\left[ {M_{ii} } \right]} & {\quad \left[ {M_{ij} } \right]} \\ {\left[ {M_{ji} } \right]} & {\quad \left[ {M_{jj} } \right]} \\ \end{array} } \right]$$

Neglecting the effect of the rotational elements of mass matrix $\left( {\frac{2}{3}m_{c} r_{c}^{2} } \right)$ resulted by the small radius of atoms $\left( {r_{c} = 2.75 \times 10^{ - 5} {\text{ {\AA}}}} \right)$, the elemental mass matrix can be described by the following relation:

19 $$\left[ {M_{ii} } \right] = \left[ {M_{jj} } \right] = \left[ {\begin{array}{*{20}c} {m_{c} /3} & {\quad 0} & {\quad 0} & {\quad 0} & {\quad 0} & {\quad 0} \\ 0 & {\quad m_{c} /3} & {\quad 0} & {\quad 0} & {\quad 0} & {\quad 0} \\ 0 & {\quad 0} & {\quad m_{c} /3} & {\quad 0} & {\quad 0} & {\quad 0} \\ 0 & {\quad 0} & {\quad 0} & {\quad 0} & {\quad 0} & {\quad 0} \\ 0 & {\quad 0} & {\quad 0} & {\quad 0} & {\quad 0} & {\quad 0} \\ 0 & {\quad 0} & {\quad 0} & {\quad 0} & {\quad 0} & {\quad 0} \\ \end{array} } \right]$$

and $\left[ {M_{ij} } \right] = \left[ {M_{ji} } \right] = \left[ 0 \right]_{6 \times 6}$. Besides, two type of stiffness matrices are considered. In the first type, the matrices is related to the beam elements, which is as follows:

20 $$\left[ K \right]_{b}^{e} = \left[ {\begin{array}{*{20}c} {\left[ {K_{ii} } \right]} & {\quad \left[ {K_{ij} } \right]} \\ {\left[ {K_{ji} } \right]} & {\quad \left[ {K_{jj} } \right]} \\ \end{array} } \right]$$

where

21 $$\left[ {K_{ii} } \right] = \left[ {\begin{array}{*{20}l} {EA/L} \hfill & {\quad 0} \hfill & {\quad 0} \hfill & {\quad 0} \hfill & {\quad 0} \hfill & {\quad 0} \hfill \\ 0 \hfill & {\quad 12EI_{x} /L^{3} } \hfill & {\quad 0} \hfill & {\quad 0} \hfill & {\quad 0} \hfill & {\quad 6EI_{x} /L^{2} } \hfill \\ 0 \hfill & {\quad 0} \hfill & {\quad 12EI_{y} /L^{3} } \hfill & {\quad 0} \hfill & {\quad - 6EI_{y} /L^{2} } \hfill & {\quad 0} \hfill \\ 0 \hfill & {\quad 0} \hfill & {\quad 0} \hfill & {\quad GJ/L} \hfill & {\quad 0} \hfill & {\quad 0} \hfill \\ 0 \hfill & {\quad 0} \hfill & {\quad - 6EI_{y} /L^{2} } \hfill & {\quad 0} \hfill & {\quad 4EI_{y} /L} \hfill & {\quad 0} \hfill \\ 0 \hfill & {\quad 6EI_{x} /L^{3} } \hfill & {\quad 0} \hfill & {\quad 0} \hfill & {\quad 0} \hfill & {\quad 4EI_{x} /L} \hfill \\ \end{array} } \right]$$

22 $$\left[ {K_{ij} } \right] = \left[ {\begin{array}{*{20}l} { - EA/L} \hfill & {\quad 0} \hfill & {\quad 0} \hfill & {\quad 0} \hfill & {\quad 0} \hfill & {\quad 0} \hfill \\ 0 \hfill & {\quad - 12EI_{x} /L^{3} } \hfill & {\quad 0} \hfill & {\quad 0} \hfill & {\quad 0} \hfill & {\quad 6EI_{x} /L^{2} } \hfill \\ 0 \hfill & {\quad 0} \hfill & {\quad - 12EI_{y} /L^{3} } \hfill & {\quad 0} \hfill & {\quad - 6EI_{y} /L^{2} } \hfill & {\quad 0} \hfill \\ 0 \hfill & {\quad 0} \hfill & {\quad 0} \hfill & {\quad - GJ/L} \hfill & {\quad 0} \hfill & {\quad 0} \hfill \\ 0 \hfill & {\quad 0} \hfill & {\quad 6EI_{y} /L^{2} } \hfill & {\quad 0} \hfill & {\quad 2EI_{y} /L} \hfill & {\quad 0} \hfill \\ 0 \hfill & {\quad - 6EI_{x} /L^{3} } \hfill & {\quad 0} \hfill & {\quad 0} \hfill & {\quad 0} \hfill & {\quad 2EI_{x} /L} \hfill \\ \end{array} } \right]{ }$$

23 $$\left[ {K_{jj} } \right] = \left[ {\begin{array}{*{20}l} {EA/L} \hfill & {\quad 0} \hfill & {\quad 0} \hfill & {\quad 0} \hfill & {\quad 0} \hfill & {\quad 0} \hfill \\ 0 \hfill & {\quad 12EI_{x} /L^{3} } \hfill & {\quad 0} \hfill & {\quad 0} \hfill & {\quad 0} \hfill & {\quad - 6EI_{x} /L^{2} } \hfill \\ 0 \hfill & {\quad 0} \hfill & {\quad - 12EI_{y} /L^{3} } \hfill & {\quad 0} \hfill & {\quad - 6EI_{y} /L^{2} } \hfill & {\quad 0} \hfill \\ 0 \hfill & {\quad 0} \hfill & {\quad 0} \hfill & {\quad GJ/L} \hfill & {\quad 0} \hfill & {\quad 0} \hfill \\ 0 \hfill & {\quad 0} \hfill & {\quad 6EI_{y} /L^{2} } \hfill & {\quad 0} \hfill & {\quad 4EI_{y} /L} \hfill & {\quad 0} \hfill \\ 0 \hfill & {\quad - 6EI_{x} /L^{3} } \hfill & {\quad 0} \hfill & {\quad 0} \hfill & {\quad 0} \hfill & {\quad 4EI_{x} /L} \hfill \\ \end{array} } \right]$$

In the second type, stiffness elemental matrix is associated with the spring element and is thus expressed by,

24 $$\left[ K \right]_{s}^{e} = \left[ {\begin{array}{*{20}c} {\left[ {K_{ii} } \right]} & {\quad \left[ {K_{ij} } \right]} \\ {\left[ {K_{ji} } \right]} & {\quad \left[ {K_{jj} } \right]} \\ \end{array} } \right]$$

where

25 $$\left[ {K_{ii} } \right] = \left[ {K_{jj} } \right] = \left[ {\begin{array}{*{20}c} {\left[ A \right]} & {\quad \left[ O \right]} \\ {\left[ O \right]} & {\quad \left[ O \right]} \\ \end{array} } \right],\quad \left[ {K_{ij} } \right] = - \left[ {K_{ii} } \right]$$

in which $\left[ O \right] = \left[ 0 \right]_{3 \times 3}$ and $\left[ A \right]$ is expressed as:

26 $$\left[ A \right] = \left[ {\begin{array}{*{20}c} \alpha & {\quad 0} & {\quad 0} \\ 0 & {\quad 0} & {\quad 0} \\ 0 & {\quad 0} & {\quad 0} \\ \end{array} } \right]{ }$$

where $\alpha = k_{vdW}$ and $k_{vdW}$ shows the stiffness of springs related with the van der Waals force. The vector of nodal displacements is given as follows:

27 $$\left\{ q \right\} = \left\{ Q \right\}_{i} Cos \omega_{i} t$$

where $\left\{ Q \right\}_{i}$, $\omega_{i}$ and $t$ are the mode shape of $i{\text{th}}$ natural frequency, the $i{\text{th}}$ natural frequency and time, respectively. By substituting Eq. (27) into Eq. (17) one would arrive at:

28 $$\left( {\left[ K \right] - \omega_{i}^{2} \left[ M \right]} \right)\left\{ Q \right\}_{i} = \left\{ 0 \right\}$$

This equality is satisfied if either $\left\{ Q \right\}_{i} = \left\{ 0 \right\}$, which is not an acceptable answer, or if the determinant of the parenthesis is set to zero. Therefore:

29 $$\left| {\left[ K \right] - \omega_{i}^{2} \left[ M \right]} \right| = \left\{ 0 \right\}{ }$$

This is an eigenvalue problem which may be solved for up to find $n$ values of natural frequencies $\omega^{2}$, where $n$ is the number of DOFs.

This eigenvalue problem should be solved to obtain $\omega_{i}$. $i = 0,1, \ldots ,n$ where $n$ the total numberof DOFs.

By substituting each of the natural frequencies (eigenvalue) into Eq.$\left( {26} \right)$, the corresponding mode shape (eigenvector) is determined. The eigenvalues and eigenvectors are obtained by using the Block Lanczos method. The natural frequencies $\left( f \right)$ is defined as:

30 $${ }f_{i} = {\raise0.7ex\hbox{${\omega_{i} }$} \!\mathord{\left/ {\vphantom {{\omega_{i} } {2\pi }}}\right.\kern-0pt} \!\lower0.7ex\hbox{${2\pi }$}}{ }$$

## Results and discussion

As discussed in the preceding section, a 3D FE simulation is employed to evaluate the natural frequencies and the corresponding mode shapes of the DWSiCNCs. In this study DWSiCNCs with different geometries under different boundary conditions are modeled. For modeling the C–C, Si–C and Si–Si bonds, the 3D elastic BEAM188 element is used here. This element is based on Timoshenko beam theory which includes shear-deformation effects and considers translations degrees of freedom (DOFs) in along x, y, and z directions and rotations about the x, y, and z axis.

**Figure 2:**
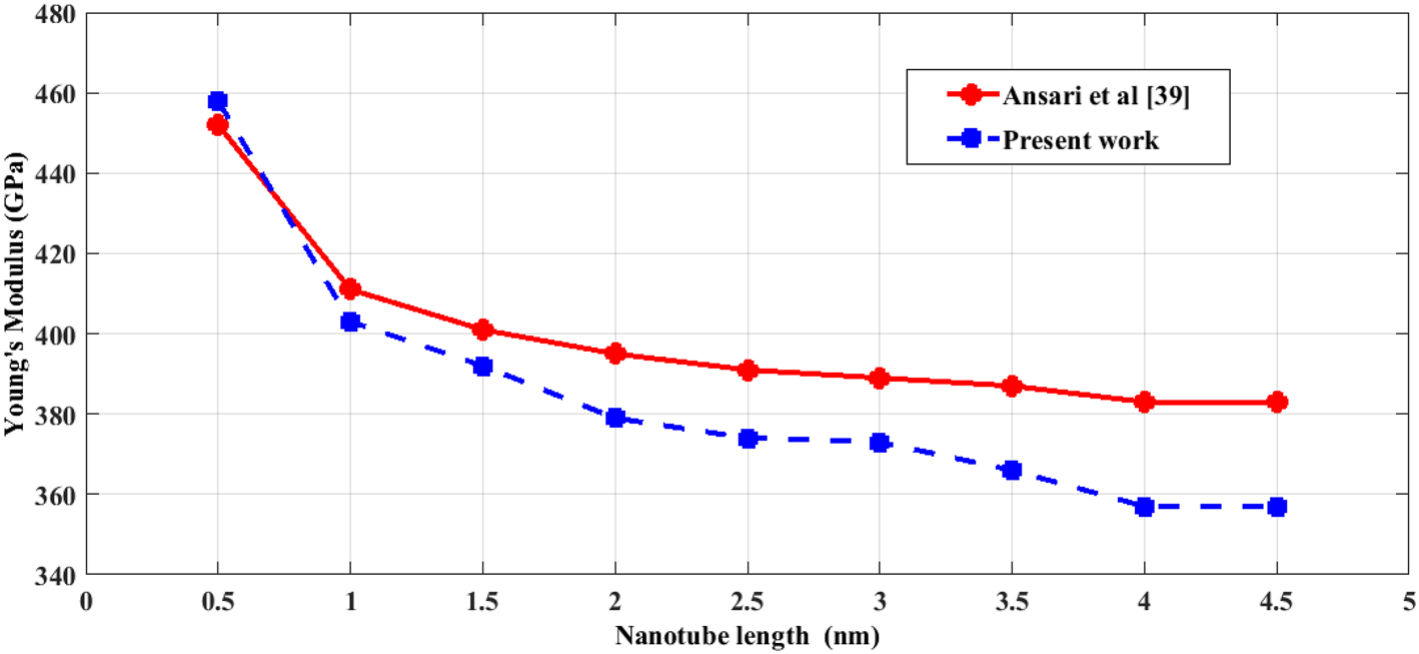
Young’s modulus of various SWCNCs against their length with small radius of 3.4 Å and apex angle of $2\alpha = 83.6^{ \circ }$.

**Figure 3:**
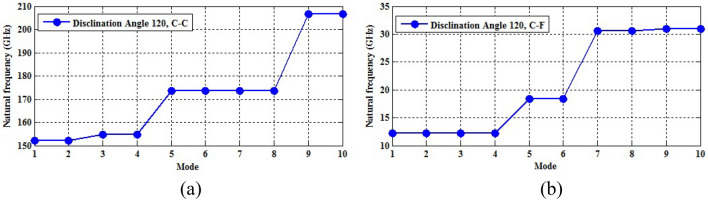
First ten natural frequencies of DWSiCNCs having the length of 20 Ǻ and disclination angles of 120° for (**a**) C–C and (**b**) C–F boundary conditions.

**Figure 4:**
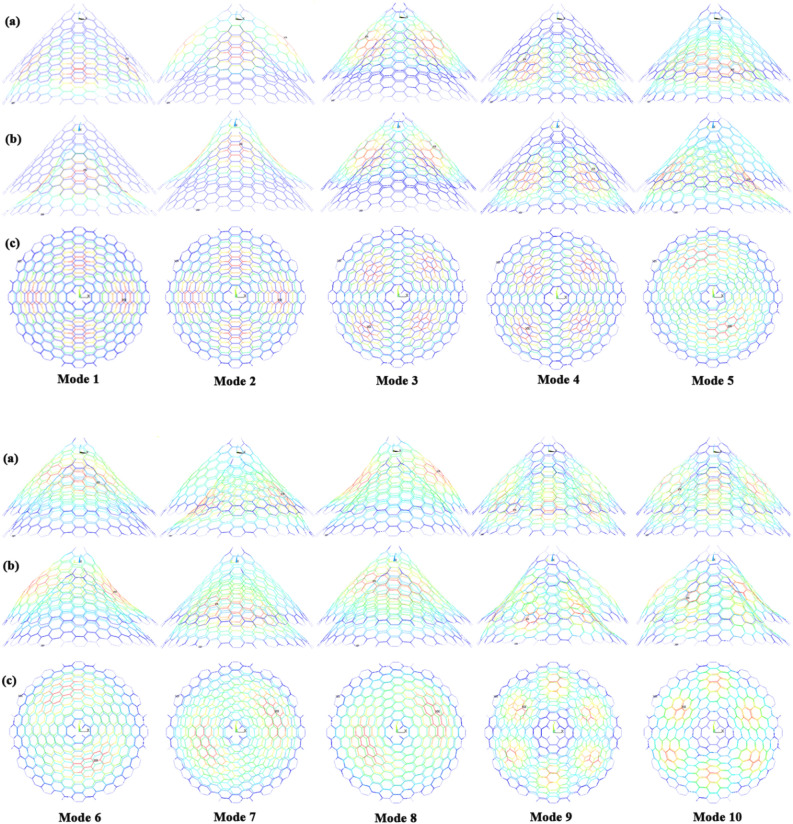
Vibrational mode shapes of the DWSiCNCs with the disclination angle of $120^{ \circ }$ and length of $20\,{\text{\AA}}$ for C–C boundary conditions.

**Figure 5:**
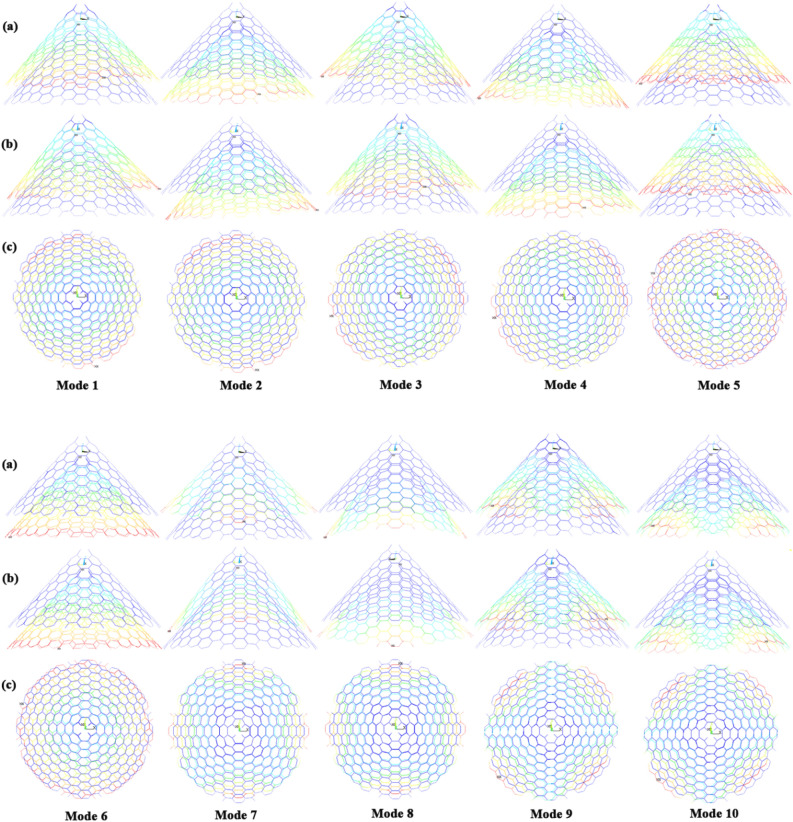
Vibrational mode shapes of the DWSiCNCs with the disclination angle of $120^{ \circ }$ and length of $20\,{\text{\AA}}$ for C–F boundary conditions.

**Figure 6:**
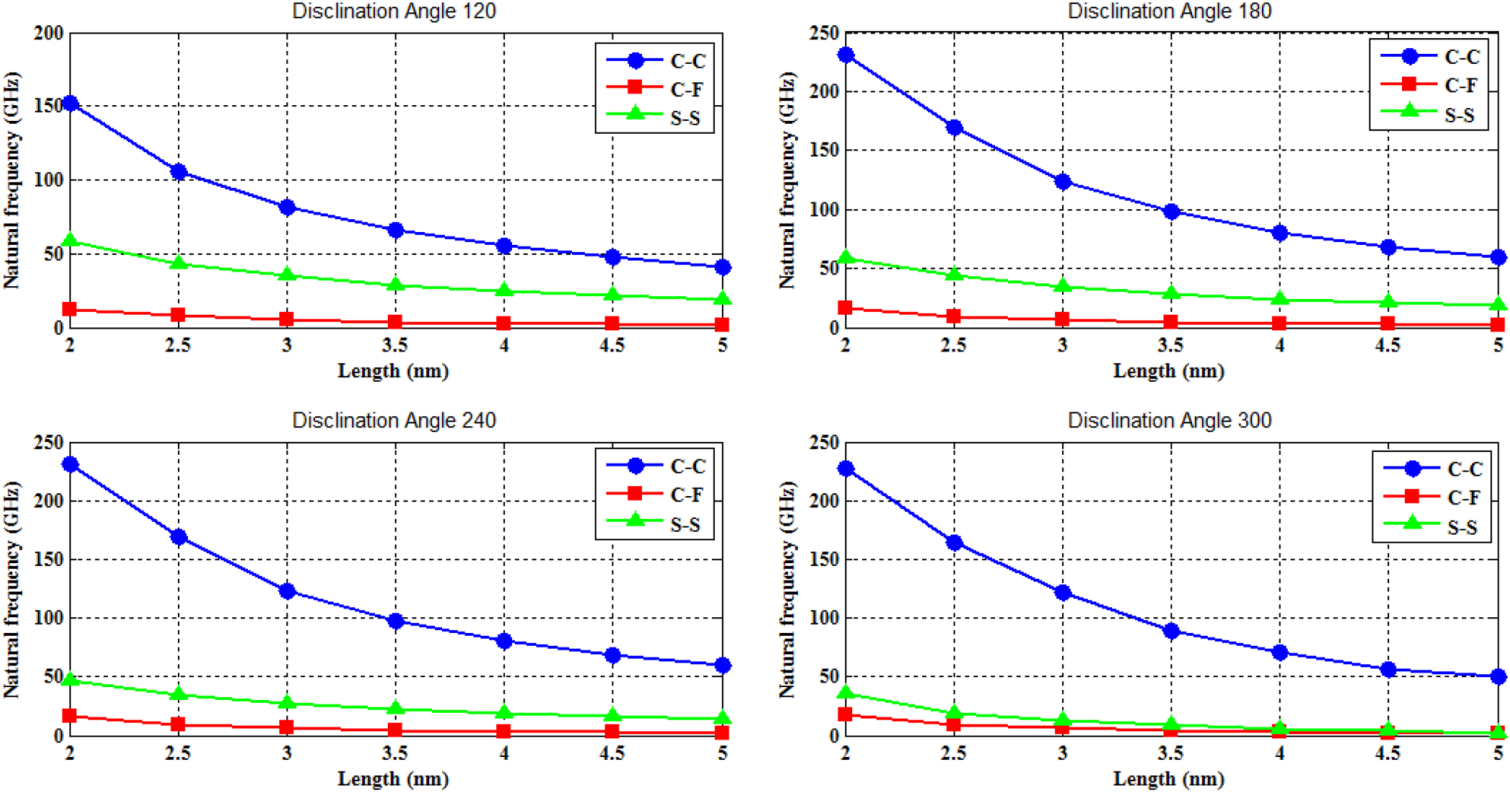
Variation of the first natural frequency of DWSiCNCs having the different disclination angle as the cone length changes for the S–S, C–F and C–C boundary conditions.

**Figure 7:**
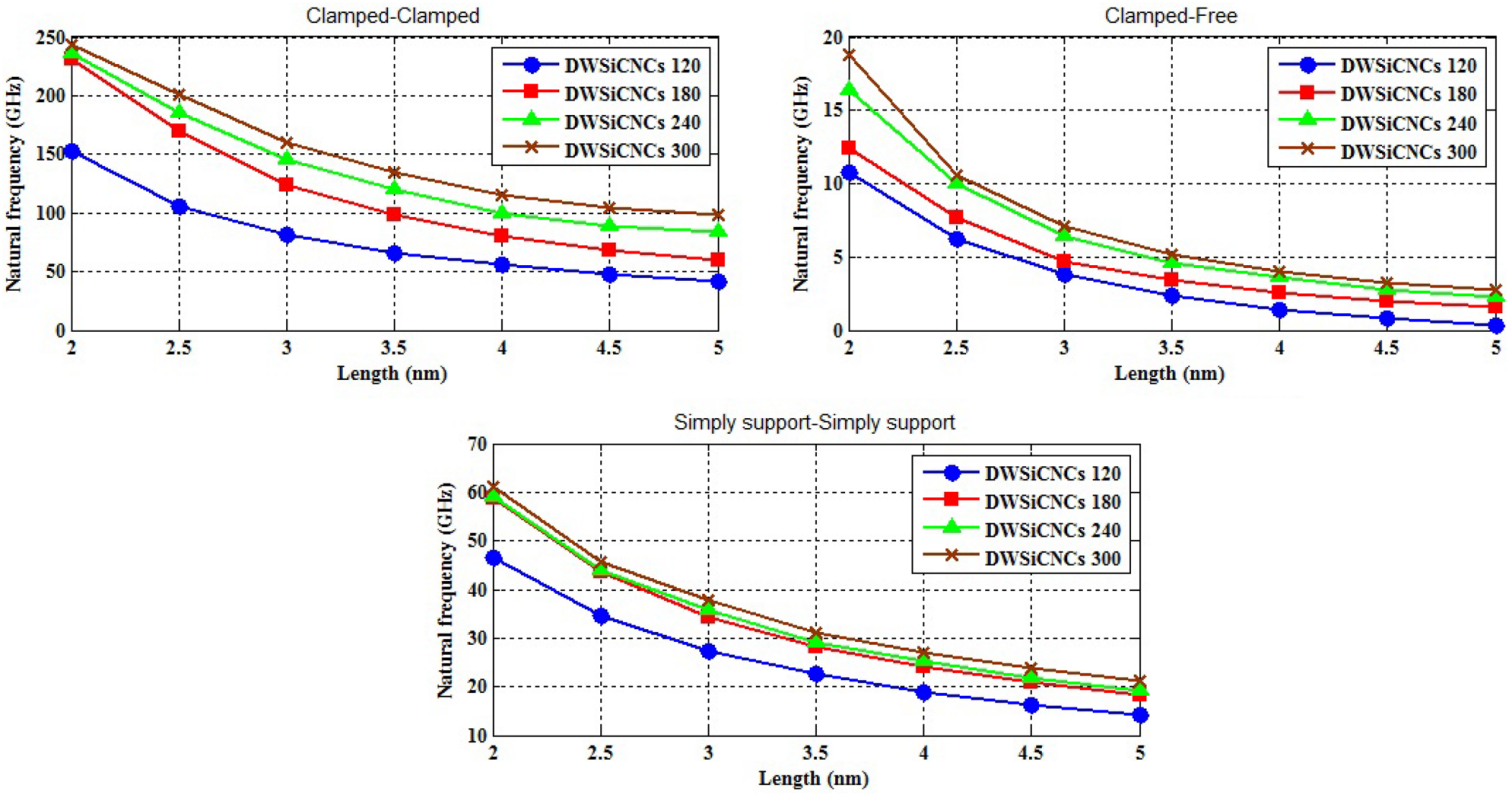
Variation of the first natural frequency of DWSiCNCs having the disclination angles of 120°,180°, 240° and 300° as the cone length changes for the different boundary condition.

**Figure 8:**
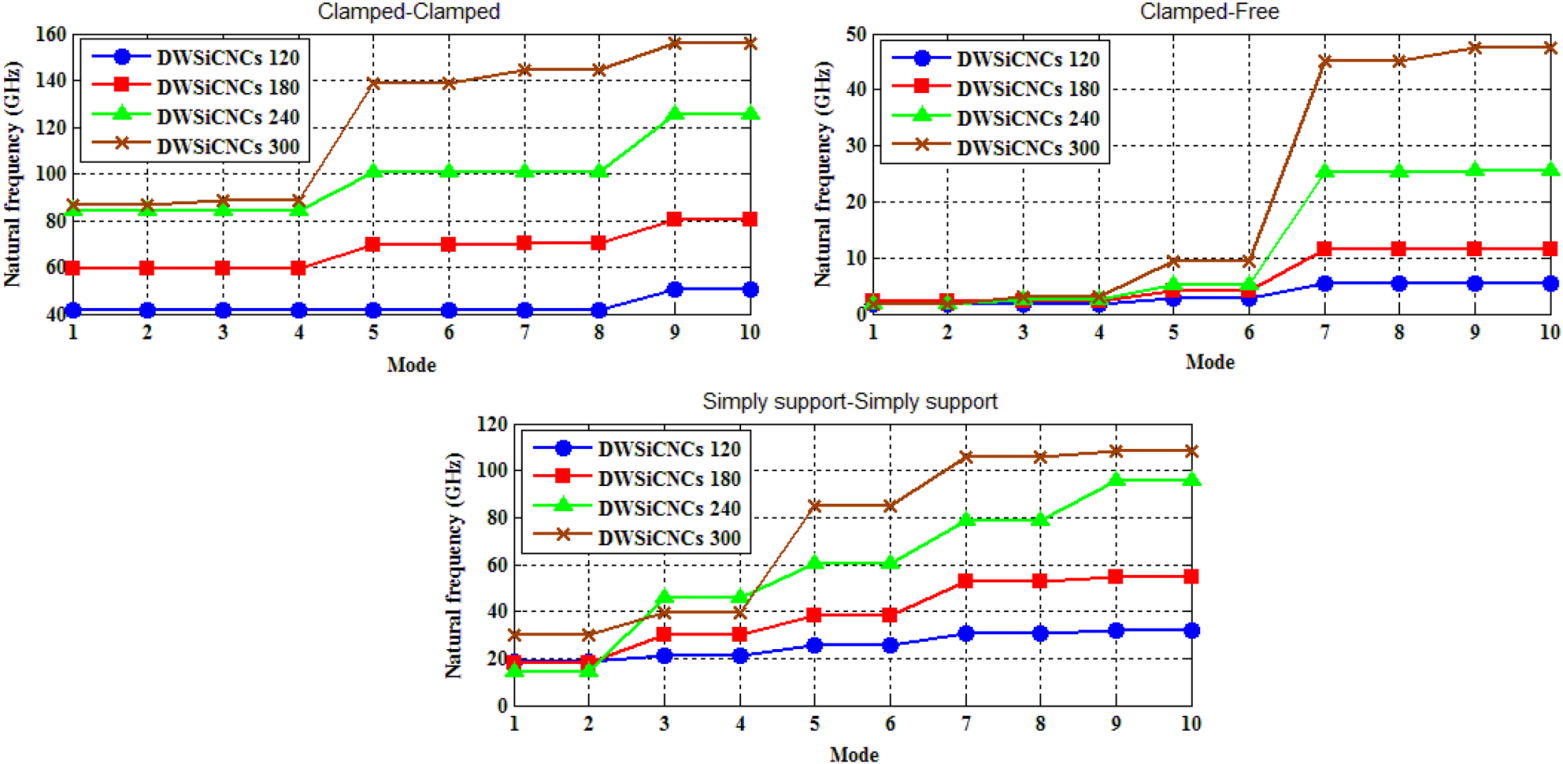
First ten natural frequencies of DWSiCNCs having the length of 50 Ǻ and disclination angles of 120°,180°, 240° and 300° for different boundary condition.

**Figure 9:**
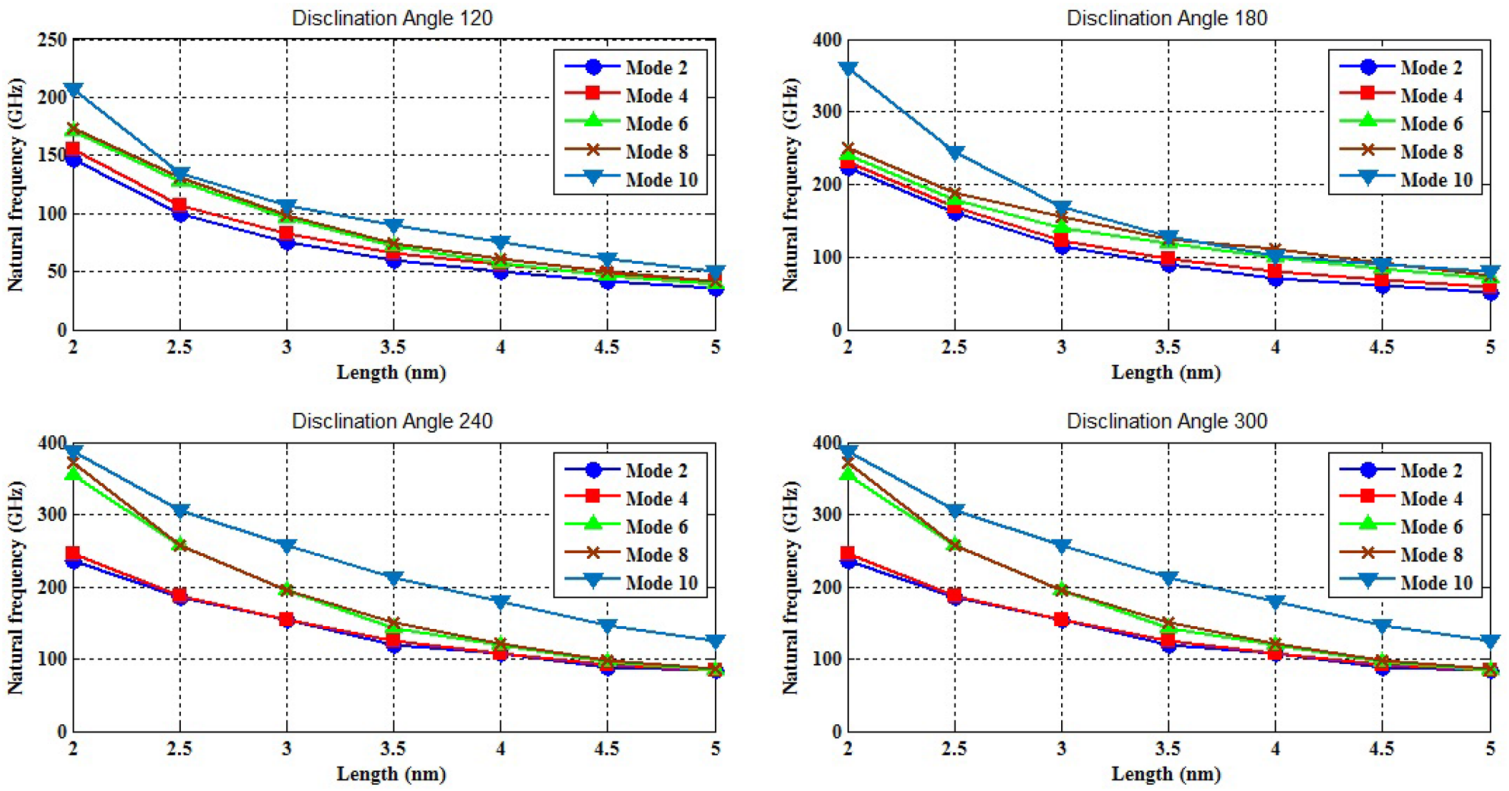
Variation of five natural frequencies of DWSiCNCs of different disclination angles for C–C boundary condition.

**Figure 10:**
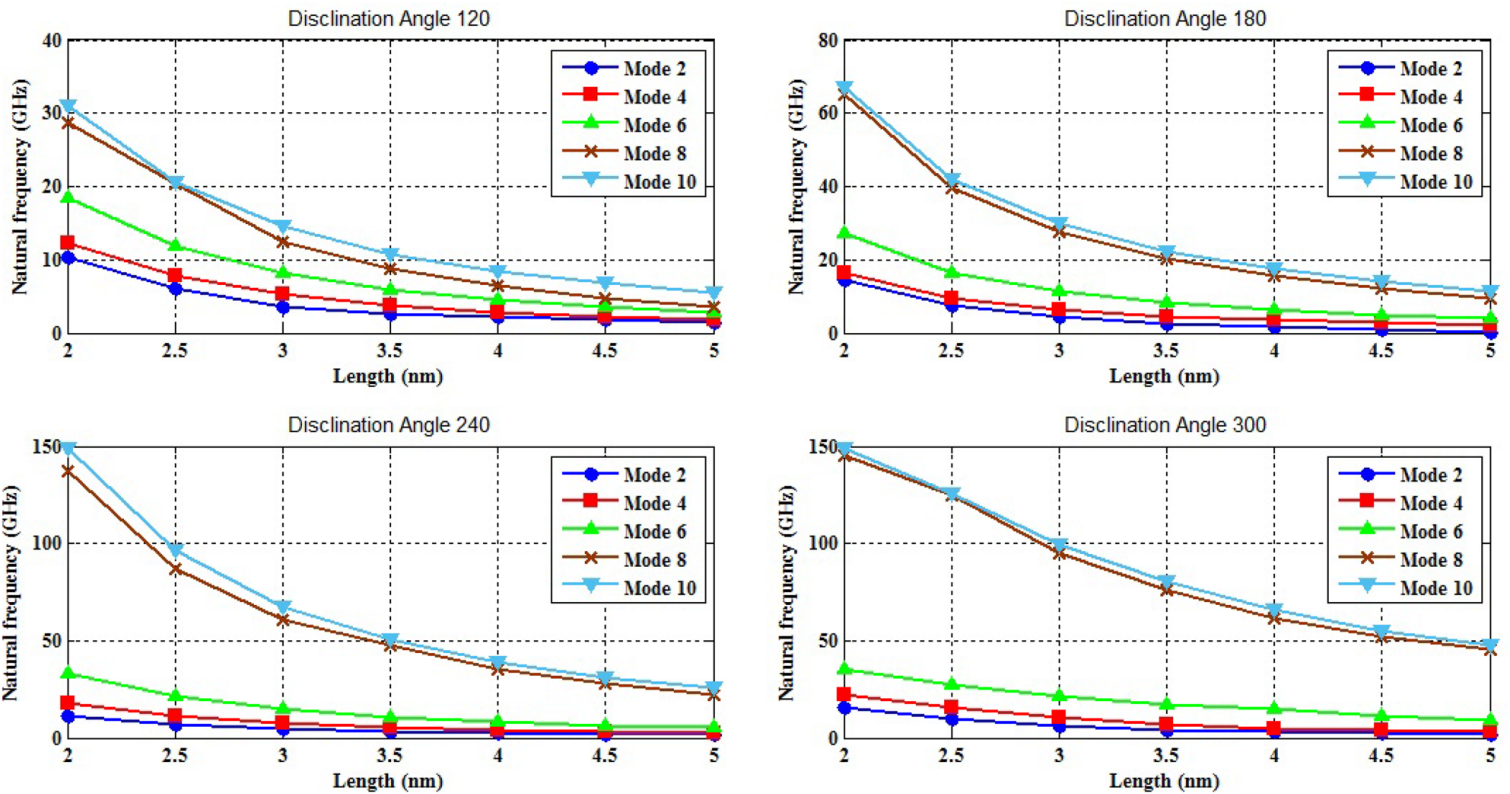
Variation of five natural frequencies of DWSiCNCs of different disclination angles for C–F boundary condition.

The naocones with the apex angles of 19.2°, 38.9°, 60° and 86.6° are considered under the simply supported-simply supported (S–S), clamped-free (C–F) and clamped–clamped (C–C) boundary conditions during the simulation. For the C–C boundary conditions, all rotational and translational movements of both ends of the nano-cone are constrained. Moreover, for the C-F boundary conditions, one end of the nano-cone is constrained from rotational and translational movements. For the S–S boundary conditions, only the translational degrees of freedom (DOFs) of the boundary nodes are constrained. Number of elements (N) of the used models are listed in Table 2. To prove the validity of the FE modeling, the results are compared with those of the MD simulations in Fig. 2. It is observed that the utilized method predicts the elastic modulus of the nano-cone with an acceptable accuracy. The precision of the method reduces by increasing the nanotube length^61^. However, after the length of 4 nm, the error percentage reaches to a constant value of about 6%. The variation of the first ten natural frequencies of a DWSiCNCs with the length of 20 Ǻ and disclination angle of 120° under C–C and C–F boundary conditions are represented in Figs. 3. The modes of vibration are also demonstrated in Figs. 4 and 5. General trends of frequencies are the same for C–C and C–F boundary conditions. According to the figures, the difference between the first to fourth frequencies remains almost unchanged. Also, in this range deformation of mode shapes are the same, but the location of the deformation changes. This fact is quite evident in a, b and c views. A sudden increase is observed between fourth and fifth frequencies. For the C–C boundary condition, the natural frequency from mode 5 to mode 8 remained constant and again, from mode 8 to mode 9 increases suddenly. While, for the C–F boundary condition, the natural frequency from mode 6 to mode 7 would increase suddenly and in then remains approximately constant. Furthermore, it is found that the maximum deformation for C–F and C–C boundary conditions occur in the base circle and the middle of the nano-cone, respectively. Figure 6. illustrates the variation of the first natural frequencies of DWSiCNCs with the disclination angles of $60^{ \circ } ,\,120^{ \circ } \,,\,180^{ \circ }$ and $240^{ \circ }$ against the nano-cone length for C–C, C–F, and S–S boundary conditions. As the results show, the natural frequency decreases with increasing DWSiCNCs length for all of the disclination angles. The natural frequencies of the DWSiCNCs with CC Boundary conditions are higher than CF and SS boundary conditions. It is also observed that the variation of the frequencies of the DWSiCNCs with the disclination angle of $120^{ \circ }$ is smaller than other disclination angles. The curves associated with the frequencies of the C-F and S–S DWSiCNCs tend to converge by increasing the nano-cone length. This is especially observable for the larger disclination angles. Moreover, considering the reduction of the difference between the curves associated with all of the boundary condition, it can be deduced that the effect of boundary conditions would be decreased by increasing the nano-cone length. Considering the higher reduction rate of the frequency at smaller length, one can conclude that the effect of length variation on the DWSiCNC frequency is more significant at smaller length. For example, the frequency of the C–C nano-cone with the disclination angles of 240° and $L = 2\,{\text{nm}}$ is $236.35\,{\text{GHz}}$ which is $21.47\,\%$ larger than that of the same nano-cone with the length of $L = 2.5\,{\text{nm}}\left( {185.62\,{\text{GHz}}} \right)$. Likewise, the difference between the magnitude curve at the point corresponding to the frequencies to $L = 4.5\,{\text{nm}}$ and $L = 5\,{\text{nm}}$ is $5.97\,{\% }$. Furthermore, for the nano-cone with the same disclination angle under the S–S boundary condition, the associated values are obtained as $26.1\,{\% }$ and $12.29\,{\% }$. Similarly, these values for the C-F boundary condition becomes $41.69\,{\% }$ and $20.1\,{\% }$. Figure 7. illustrates the variations of the first natural frequencies of the C–C, C–F and S–S DWSiCNCs versus the nano-cone length for different disclination angles. It can be observed that the fundamental natural frequency of DWSiCNCs increases by increasing the disclination angle. The maximum fundamental frequency was obtained for all types of boundary condition at a disclination angle of $300^{ \circ }$ and minimum value happened at $120^{ \circ }$. However, it would be decreased by increasing the nano-cone length for all of the disclination angle and boundary conditions. The natural frequency of a structure can be approximated by the relation of ${\text{f}} = 1/2\pi \sqrt {K/M}$, where $K$ and $M$ are the general stiffness and mass of the strcuture. According to this relation, decreasing the natural frequency can be related to decreasing the ratio of the stiffness to the mass. Therefore, it can be concluded that increasing the length results in decreasing the ratio of $K/M$. Also, it is shown that the slope of the curve in the small length range is much greater than that in the high length range. The difference between the curves is more significant for the C–C DWSiCNTs. However, for the other two boundary conditions, the effect of the disclination angles decreases. Especially for the S–S nano-cone, the difference between the frequencies of the nano-cones with the designation angles of $180^{ \circ }$, $240^{ \circ }$ and $300^{ \circ }$ is negligible. The First ten natural frequencies of DWSiCNCs under C–C, C–F, and S–S boundary conditions are plotted in Fig. 8 versus mode number. It is observed that the frequency does not experience significant variation between the first and fourth modes. However, the frequency enhances significantly between the 4th and 5th and then between 8 and 9th modes for C–C boundary conditions. For C–F and S–S boundary conditions, variation of frequency occurs between the 4th and 5th and then between 6 and 7th modes. Moreover, it is found that, the largest frequencies are associated with the C–C boundary conditions. Furthermore, all of the frequencies increase by increasing the disclination angle. Comparison the curves related to different disclination angles shows that by increasing the mode number, the distance between of the natural increases. In other words, as it can be seen in Table 3, for the frequencies of a C-F DWSiCNCs, the difference percentage of natural frequency increases at larger modes. For a vibrational system, the eigenvalues and eigenvectors associated with the eigenvalue problem have significant physical meanings. The natural frequencies $({\upomega }_{{\text{i}}})$ of the nano-cone is equal to the square root of the eigenvalues. The natural frequencies are usually arranged in increasing order of magnitude, that is, ${\upomega }_{1}\le {\upomega }_{2}\le {\upomega }_{3}\le \dots \le {\upomega }_{{\text{n}}}$. The eigenvectors are referred to as modal vectors. Each modal vector represents physically the shape of a normal mode, a certain pattern of motion in which all masses move harmonically with the same natural frequency associated with this modal vector. Figures 9 and 10 illustrate the variation of 2nd, 4th, 6th, 8th and 10th frequencies of DWSiCNCs with the disclination angles of $60^{ \circ } ,\,120^{ \circ } \,,\,180^{ \circ }$ and $240^{ \circ }$ versus the nano-cone length under C–C and C–F boundary conditions, respectively. From this figure, it can be seen that increasing the length results in decreasing the natural frequencies, while that with increasing the disclination angle, the natural frequencies increase. The natural frequency curves associated with different disclination angles converge at higher lengths. For instance, differences between in the 6th and 10th natural frequencies of the DWSiCNCs with the $L = 2\,{\text{nm}}$ and disclination angles of $120^{ \circ }$, $180^{ \circ }$,$240^{ \circ }$ and $300^{ \circ }$ are around $40.66\,{\% }$, $59.33\,{\% }$, $77.53\,{\% }$ and $77.53\,{\% }$, respectively. However, variation in the natural frequencies with s $L = 5\,{\text{nm}}$ increased around $48.26\,{\% }$, $64.64\,{\% }$, $79.59\,{\% }$ and $80.18\,{\% }$ at modes 6 and 10.

**Table 2:** Number of elements (N) in different disclination angles.

Length (Å)	N in different disclination angles			
	$120^{ \circ }$	$180^{ \circ }$	$240^{ \circ }$	$300^{ \circ }$
20	1048	552	328	132
25	1632	918	516	220
30	2360	1332	748	322
35	3256	1930	1008	444
40	4272	2340	1340	598
45	5392	2988	1684	764
50	6712	3714	2088	940

**Table 3:** Comparison among frequencies (GHz) for different disclination angles under C-F.

Mode	$120^{ \circ }$	$180^{ \circ }$	${\text{Diff}}.\left( {\% } \right)$	$180^{ \circ }$	$240^{ \circ }$	${\text{Diff}}.\left( {\% } \right)$	$240^{ \circ }$	$300^{ \circ }$	${\text{Diff}}.\left( {\% } \right)$
1	1.8275	2.2271	17.94	2.2271	1.5933	5.95	1.5933	1.7976	11.37
3	1.8275	2.2274	17.95	2.2274	2.4738	9.96	2.4738	3.0395	18.61
5	2.8692	4.0393	28.96	4.0393	5.2296	22.76	5.2296	9.4388	44.60
7	5.538	11.423	51.45	11.423	25.378	54.98	25.378	45.198	43.85
9	5.5454	11.423	56.39	11.423	25.619	55.41	25.619	47.609	46.20

### Comparison and validation of the current work

Some works has been conducted to investigate the vibrational properties and behavior of nanoscale structures, specifically double-walled silicon carbide nano-cones and single- and multi-walled carbon nanotubes^62–64^. Finite element models and molecular structure modeling were employed to simulate the dynamic behavior of these nanostructures. The effects of dimensions, boundary conditions, defects, and attachment of nanoparticles on the vibrational characteristics were investigated. These studies revealed that the stiffness, frequency response, and resonance behavior of the nanostructures are influenced by factors such as length, apex angles, disclination angles, and bond orientation. The simulation results in the current work are compared with those obtained from molecular dynamics (MD) simulations to validate the accuracy of the FE model^65^. The analysis revealed that the natural frequencies of DWSiCNCs varied with the nano-cone length and disclination angle under different boundary conditions. Notably, an increasing trend of natural frequencies was observed with increasing disclination angle^66^. The mode shapes exhibited distinct deformation patterns, with variations depending on the boundary conditions applied. The FE simulation provided valuable insights into the dynamic behavior of DWSiCNCs and offered a complementary perspective to the MD simulations^67,68^.The validation of the FE model against MD simulations confirmed its accuracy and reliability in predicting the dynamic response of DWSiCNCs. The obtained results highlighted the influence of geometric parameters and boundary conditions on the natural frequencies and mode shapes, with implications for the design and engineering of nanoscale devices and structures. Further investigations can build upon these findings to explore additional aspects of DWSiCNCs and extend the understanding of their mechanical behavior^69,70^.

### Real-world applications of DWSiCNCs

The investigation of the effects of geometrical parameters, such as length and disclination angle, on the natural frequencies of Double-Walled Silicon Carbide Nanotubes (DWSiCNCs) provides valuable insights that can have practical implications in real-world applications. Here are some potential elaborations on the practical implications of these findings:

- *Design and Optimization* Understanding how geometrical parameters affect the natural frequencies of DWSiCNCs enables researchers and engineers to design and optimize nanotube structures for specific applications. By manipulating the length and disclination angle, it becomes possible to tailor the natural frequencies of DWSiCNCs to desired ranges. This knowledge can be applied in the design of nanoelectromechanical systems (NEMS), sensors, resonators, and other nanoscale devices where precise control over the frequency response is crucial.
- *Mechanical Characterization* The relationship between geometrical parameters and natural frequencies provides a means for non-destructive mechanical characterization of DWSiCNCs. By measuring the natural frequencies of nanotubes, it becomes possible to extract information about their length and disclination angle. This can be particularly useful for quality control, assessing structural integrity, or characterizing the properties of individual nanotubes in nanocomposite materials, where the mechanical behavior and performance are critical.
- *Sensing and Detection* The sensitivity of DWSiCNCs to changes in geometrical parameters, as reflected in the variations in natural frequencies, can be harnessed for sensing and detection applications. For instance, alterations in the length or disclination angle of DWSiCNCs due to external stimuli (e.g., strain, temperature, or gas adsorption) can be detected by monitoring changes in their natural frequencies. This capability can be utilized in various sensing platforms, such as nanomechanical sensors, resonant devices, or nanoscale transducers, for applications such as gas sensing, strain gauges, or environmental monitoring.
- *Fundamental Research* Investigating the influence of geometrical parameters on the natural frequencies of DWSiCNCs contributes to the fundamental understanding of nanoscale mechanics and the behavior of carbon-based nanotubes. This knowledge not only advances the knowledge base in nanoscience and nanotechnology but also provides insights that can be extended to other nanoscale systems with similar structural characteristics. The findings may inspire further research and exploration of novel nanomaterials, nanostructures, and their applications in various fields.

## Conclusion

Using FE method, the vibrational behavior of the DWSiCNCs with different disclination angles was characterized herein. Furthermore, different boundary conditions, including C–C, C–F and S–S were applied to the nano-cones. The mode shapes of DWSiCNCs were obtained for CF and CC boundary conditions. Comparing the mode shapes with the CF and CC boundary conditions, it was deduced that the maximum deformation occur in the base circle and the middle of the nano-cones, respectively. The effects of geometrical parameters such as length and disclination angle on the natural frequency or DWSiCNCs were investigated. The numerical results indicate that increasing the mode number and the disclination angle results in increasing the natural frequency, while natural frequency decreases by increasing the length. Overall, these studies contribute to the understanding of nanoscale vibrational properties and provide insights into the potential applications of these structures in sensing and other fields.

## Data Availability

The datasets used and/or analyzed during the current study available from the corresponding author on reasonable request.
